# Tailoring morphological and electrical properties of nanoplate-ZnO varistors *via* sintering temperature†

**DOI:** 10.1039/d5ra01534k

**Published:** 2025-06-12

**Authors:** Huy Nguyen Trung, Trang Nguyen Van, Kieu Anh Vo Thi, Hong Cao Thi, Xuyen Nguyen Thi, Tuan Anh Nguyen, Tuan Anh Nguyen, Lam Tran Dai, Chinh Tran Van, Duy Lai Van, Duong La Duc, Tham Do Quang

**Affiliations:** a Institute for Tropical Technology, Vietnam Academy of Science and Technology 18 Hoang Quoc Viet Street, Cau Giay District Hanoi City Vietnam dqtham@itt.vast.vn; b Graduate University of Science and Technology (GUST), Vietnam Academy of Science and Technology 18 Hoang Quoc Viet Street Cau Giay Hanoi Vietnam; c Institute of Chemistry and Materials 17 Hoang Sam, Nghia Do Cau Giay Hanoi Vietnam duc.duong.la@gmail.com; d Institute of Materials Science, Vietnam Academy of Science and Technology 18 Hoang Quoc Viet Street, Cau Giay District Hanoi City Vietnam

## Abstract

In this study, ZnO nanoplates (crystallite size: 100 nm, thickness: 15 nm) were synthesized *via* a hydrothermal route. Varistors were then fabricated using these ZnO nanoplates incorporated with five oxide dopants (Bi_2_O_3_, Sb_2_O_3_, MnO_2_, Co_3_O_4_, and Cr_2_O_3_) and sintered at 1000, 1100, and 1200 °C. A control varistor sample using micro-sized ZnO was also prepared. The effects of sintering temperature on the structural, mechanical, and electrical properties of ZnO-based varistors were systematically studied. Increasing the sintering temperature from 1000 °C to 1200 °C enlarged the grain size (1.7–6.8 μm), enhanced hardness (200–280 HV), and resulted in 17–19% shrinkage. At 1100 °C, the varistor achieved a balance of high nonlinearity (*α* = 48.5), low leakage current (*J*_L_ = 9.7 μA cm^−2^), and high breakdown threshold (*E*_b_ = 689 V mm^−1^). Impedance analysis showed a resistive–capacitive transition at higher frequencies, while grain boundary resistivity at low frequencies (10^6.5^–10^8^ Ω m) aligned with DC resistivity at the low applied electric fields. These results highlight the advantages of ZnO nanoplates in enhancing the electrical performance of varistors, making them promising for high-voltage applications.

## Abstract 2

ZnO nanoplate-based varistor doped with Bi_2_O_3_, Sb_2_O_3_, MnO_2_, Co_3_O_4_, and Cr_2_O_3_, sintered at 1100 °C, shows optimal properties: uniform grains, high hardness, low leakage current, and high nonlinearity.

## Graphical abstract

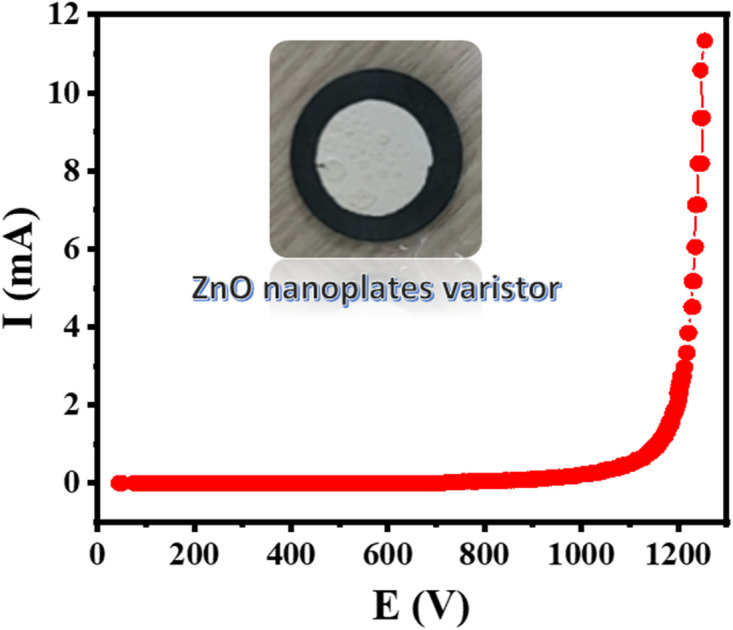

## Introduction

Varistors, or voltage-dependent resistors, are widely used in both low-power electronic devices and high-power electrical distribution systems to protect circuits from transient voltage surges through their nonlinear current–voltage (*I*–*V*) characteristics.^1–5^ Various materials such as SiC, TiO_2_, SnO_2_, WO_3_, and SrTiO_3_ have been explored as base materials for varistors with nonlinear voltage–current (*V*–*I*) characteristics. SiC varistors were among the earliest used but have low nonlinearity coefficient (*α* < 10), and require internal spark gaps due to high leakage current. TiO_2_-based varistors offer high dielectric constant and surge absorption capacity, but suffer from low breakdown voltage and nonlinearity, limiting them in low-voltage surge protection applications. WO_3_-based varistors also operate at low-voltage applications due to their very low breakdown voltage (6–10 V mm^−1^). They are suitable for ceramic capacitor. SrTiO_3_ offers advantages for low-voltage varistor-capacitor dual function, owing to its high dielectric constant and nonlinear behavior. SnO_2_-based varistors offer good thermal stability and nonlinearity but still exhibit a higher residual voltage ratio. However, they exhibit relative higher residual voltage ratio (>2) than ZnO-based varistors (∼1.7) (residual voltage ratio is defined as the ratio of the residual voltage under high impulse current and the voltage under the 1 mA DC current). Currently, SnO_2_ based varistors are in the developing stage. Meanwhile, ZnO-based varistors dominate commercial applications due to their high nonlinear coefficient (*α* > 30), excellent energy-handling capability, and cost-effective mass production.^3^

The breakdown voltage of a ZnO-based varistor is primarily influenced by the average ZnO grain size. For high-voltage applications, a small average grain size (<10 μm) is required, whereas for low-voltage applications, a larger grain size (>30 μm) is preferred.^6^ The key electrical characteristics of varistors include the nonlinear coefficient and the breakdown voltage. The nonlinear properties of ZnO-based varistors depend not only on the material composition and processing methods but also on the microstructural uniformity of the sintered body. Microstructural inhomogeneities in the sintered varistor can lead to dielectric breakdown and failure. Under high-energy pulses, localized thermal runaway may cause puncturing or even melting.^7^

Numerous studies have shown that reducing the particle size of raw materials enhances microstructure uniformity and improves electrical properties, including a higher nonlinear coefficient and lower leakage current. Chemical methods, including sol–gel synthesis, co-precipitation, and wet chemical precipitation, have been widely explored for the preparation of ZnO-based nanopowders. These techniques allow for precise control over particle morphology and facilitate homogeneous additive distribution, which is critical for achieving stable electrical performance in varistors. For instance, the sol–gel method has been used to produce low-voltage ZnO-based varistors with refined grain structures, resulting in optimized breakdown voltage characteristics. Similarly, ZnO nanopowders synthesized *via* co-precipitation demonstrate superior aging resistance compared to conventional micro-sized ZnO powders.^8–12^

Compared to chemical method, mechanical milling is simpler and more cost-effective approach for homogenizing fine particles in varistor ceramics.^13^ J. Zhu *et al.*^14^ demonstrated that intensive milling effectively reduced ZnO powder inhomogeneity, yielding an optimal average grain size of 5.1 μm, and enhancing varistor performance. More recently, Li *et al.*^15^ reported a breakthrough in milling efficiency by combining ball milling with sand milling, achieving a grinding rate 6.68 times faster than traditional methods, and significantly increasing the nonlinear coefficient from 29.1 to 47.

Boumezoued *et al.*^16^ investigated the synthesis and characterization of ZnO-based nanopowders, with a focus on how sintering temperature affects the performance of ZnO–Bi_2_O_3_ varistors. In this study, the authors examined a simplified varistor system composed solely of nano-ZnO and Bi_2_O_3_ as the primary additive. Their preliminary results highlighted the influence of sintering conditions on microstructure development, densification behavior, and electrical properties. This study offered valuable insights into the role of Bi_2_O_3_ in grain growth and varistor performance, laying the groundwork for further research on additional dopants to improve electrical characteristics.

P. K. Roy aimed to enhance the electrical properties of ZnO-based varistors by utilizing ultra-fine ZnO powders (3–10 nm). ZnO-based varistors were fabricated with Bi_2_O_3_, Sb_2_O_3_, CoO, MnO_2_, Cr_2_O_3_, and SiO_2_ as dopants and sintered at temperatures ranging from 850 °C to 1150 °C. The sintered varistors had a density of ∼99% (relative to the theoretical value), a grain size of ∼5–7 μm, and a maximum breakdown voltage of 292 V mm^−1^.^8^

S. Anas synthesized nano-platelet ZnO particles with an average size of 50 nm *via* the sol–gel technique and used them to fabricate nano-ZnO varistors. The SEM images of the calcined ZnO varistor powders were shown that the crystalline ZnO was only partially formed, with platelet like morphology. The results showed that raising the sintering temperatures in the range of 850, 950, 1050, and 1150 °C increased the grain size of ZnO from 2 to 8 μm, while the breakdown voltage decreased from 557 V to 323 V, and the highest nonlinearity (*α* = 36) was observed at a sintering temperature of 1050 °C. Compared to conventional spray-dried varistors, the nano-frame varistors exhibited higher breakdown voltage and nonlinearity.^17^

Pillai *et al.*^18^ prepared nano ZnO using mixed precursor method (MPR) and nanoZnO based varistor. A comparison was made between a commercial varistor and the one made from the nano ZnO powder sintered at 1050 °C for 2 hours. The results showed that the varistor made form nano ZnO exhibited higher linear coefficient (*α* = 33 ± 3) and breakdown voltage (*E* = 941 V mm^−1^) compared to the commercial varistor (*α* = 28 ± 3; *E* = 507 V mm^−1^).

Liu *et al.*^19^ studied the preparation and characterization of ZnO varistor ceramics using nano-sized ZnO powders *via* the solid-state reaction method. The study demonstrated significant improvements in the voltage gradient (803 V mm^−1^) and energy absorption capability (400 J cm^−3^) compared to conventional varistors. These enhancements were attributed to changes in micromorphology, as SEM results revealed a reduction in average grain size from 17.33 μm to 3.63 μm.

These findings underscore the growing potential of nano-ZnO varistors over their micro-ZnO counterparts. Control of the grain size is important for tailoring the breakdown voltage of varistor ceramics.^7^ Theoretically, the breakdown voltage is determined by the voltage drop across the grain boundaries. Reducing grain size increases, the number of boundaries per unit length, and thus leads to a higher breakdown voltage in varistors. By leveraging chemical synthesis or advanced milling techniques, researchers have been able to control grain size distribution, enhance densification behavior, and optimize the electrical performance of varistors. However, further comparative studies are required to fully evaluate the advantages of nano-ZnO varistors under varying processing and sintering conditions.

To tailor and enhance the nonlinear electrical behavior of ZnO-based varistors, each study typically employs its own specific dopant system. As a result, both the types and concentrations of additives vary widely across the literature. However, certain functional roles of the main dopants are commonly recognized. Bi_2_O_3_ plays a key role as a liquid-phase sintering aid and grain boundary modifier and the varistor effect. Sb_2_O_3_ contributes to the formation of a spinel-type secondary phase (typically Zn_7_Sb_2_O_12_), which acts as a grain growth inhibitor and helps stabilize the microstructure. Other oxides (Co_3_O_4_, MnO, and Cr_2_O_3_) are commonly used to modify the electronic structure at the grain boundaries. These dopants influence the barrier height and the density of defect states, thereby enhancing nonlinearity and reducing leakage current.

In this study, ZnO nanoplates were synthesized using a simple hydrothermal method. The nano-ZnO was utilized for the fabrication of varistors by incorporating the same dopants (Bi_2_O_3_, Sb_2_O_3_, Co_3_O_4_, MnO_2_, Cr_2_O_3_) *via* the wet ball milling, drying, mesh sieving, pellet compaction and sintering at different temperatures. The AC impedance, DC electrical properties, and hardness of nano-ZnO-based varistors were investigated. To further evaluate the advantages of ZnO nanoplates, a comparative study was conducted using varistors fabricated from micro-ZnO with identical additive system and processing conditions. This comparison provided deeper insights into the enhanced performance of varistors employing ZnO nanoplates.

## Experimental section

### Materials

Zinc sulfate heptahydrate (ZnSO_4_·7H_2_O) and urea (CH_4_N_2_O) were obtained from Sigma-Aldrich (USA). Bismuth oxide (Bi_2_O_3_, 99.0%), antimony oxide (Sb_2_O_3_, 99.99%), chromium trioxide (Cr_2_O_3_, 99.0%), ethanol (99.8%), manganese dioxide (MnO_2_, 85%) were the reagent products of Xilong Scientific company (China). Tricobalt tetraoxide (Co_3_O_4_, 98%) was purchased from Shanghai Zhanyun Chemical company (China). ZnO (99.0%) in micro powder form with average size of 1 μm was a reagent product of Guangdong Guanghua Sci-Tech company (China). The raw materials utilized for fabricating zinc oxide varistors consisted of synthesized nano-ZnO as the primary component and oxides powders (Bi_2_O_3_, Sb_2_O_3_, Co_3_O_4_, Cr_2_O_3_, and MnO_2_) as additives. All chemicals and raw materials used in this study were of analytical-grade purity and used as received without any purification.

### Synthesis of ZnO nanoplates and evaluation of specific surface area

ZnO nanoplates were synthesized using a hydrothermal method from ZnSO_4_·7H_2_O as precursor. The fabrication process for ZnO nanoplates began by dissolving 10 mmol of ZnSO_4_·7H_2_O in 30 mL of deionized water. Subsequently, 20 mL of a CH_4_N_2_O solution (20 mmol) was added to adjust the pH to 5, and the mixture was stirred for 15 minutes. The prepared solution was then transferred to a 100 mL Teflon-lined autoclave for hydrothermal treatment at 220 °C for 24 hours. After allowing the system to cool naturally, the resulting precipitate was collected *via* centrifugation, thoroughly washed with deionized water and ethanol, and dried at 60 °C. Finally, the product underwent calcination at 600 °C to complete the formation of the nanoplate structure. To obtain a sufficient amount for six varistor samples, several batches of ZnO nanoplate powder were synthesized using the same procedure described above (0.6–0.7 g of ZnO powder per batch). The similar synthesis procedure can be found somewhere in the previous studies.^20^

Nitrogen adsorption/desorption isotherms at 77 K, measured using a Micromeritics Gemini VII instrument, were used to determine the specific surface area (SBET) and pore size distribution, with the Brunauer–Emmett–Teller (BET) method applied. The evaluated results show that ZnO nanoplate powder has a surface area of 52.42 m^2^ g^−1^. Fig. S1† also shows its pore volume and average pore size are of 0.086 cm^3^ g^−1^ and of 0.892 nm, respectively.

### Varistors ceramics fabrication

Reagent-grade raw materials were used for preparing the samples with a composition of 96.5 mol% ZnO, 1.25 mol% Bi_2_O_3_, 0.5 mol% Co_3_O_4_, 0.5 mol% Cr_2_O_3_, 0.5 mol% MnO_2_, 1.25 mol% Sb_2_O_3_. Distilled water was added to the mixture under manual stirring to form a paste in an agate mortar, zirconium ball was then added to the paste. After ball milling for 18 h, the mixture was dried at 120 °C for 12 h. Polyvinyl alcohol (PVA) was used as an adhesive, PVA with the amount of 2 wt% (compared to total oxide mass) were added to the mixture grind and sieve through a 160 μm mesh to produce the starting power. The powder was pressed into discs of 13 mm in diameter and about 1.7 mm in thickness at a pressure of 1200 kg cm^−2^. The pellets were preheated at 600 °C for 1 hour, followed by sintering at different temperatures (1000, 1100, and 1200 °C) for 2 hours in ambient condition, and finally cooled down to 100 °C for 6 h in a programmable thermal oven (Nabertherm LHTC 03/16, USA). Sintered samples were then taken out the oven, cooled down to room temperature, and polished by using an auto grinding and polishing machine (FPT-1AH, Future-Tech, Japan). The silver paste was coated on both sides of the samples with the same diameter using one type of hollow circle stickers and then heated to 600 °C for 10 min to obtain circular thin film electrodes. Cool samples were kept in PE bags at room condition for at least 24 before any characterization.

A varistor sample using micro-ZnO (with the same composition of above five oxides) was also prepared as control samples. The control samples were also prepared using the same procedure and sintered at 1100 °C.

### Characterizations

#### Shrinkage, density, and hardness

The varistor disks were obtained by compacting raw material (ZnO, additives and binder in powder form) in a die with the same pressure of 100 MPa. The green varistor disks have a density of about 62–65% in compared with that of ZnO in crystalline form.

The axial and radial shrinkage (*Δ*_axi_ and *Δ*_dia_, %) was evaluated from the differences of both the height (*H*) and the diameter (*D*) of a varistor sample before (*H*_o_, *D*_o_) and after sintering process that was under the fully cooled condition (*H*, *D*), by using eqn (1) and (2).1
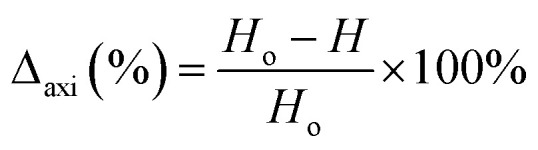
2
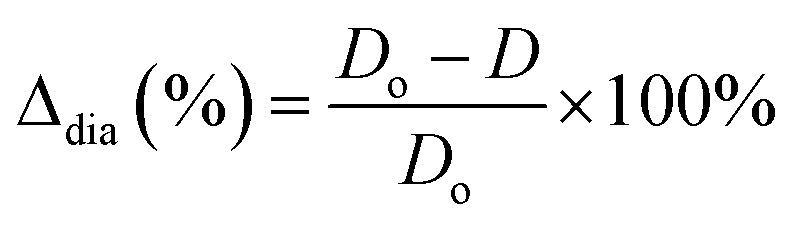


The density of a varistor was tested on a balance (AS220.R2+, Radwag, Poland) equipped with a solid density function (F6) using distilled water as medium at 25 °C.

The hardness of the varistor samples was measured by using a NEXUS 7700 Vickers test (Innovatest Holland). The resulting indent diagonal lengths were measured to calculate the Vickers Pyramid Number (HV, or [MPa]), the hardness of a sample was calculated by using the eqn (3), and averaged from at least 5 different locations on a sample under test:^21^3
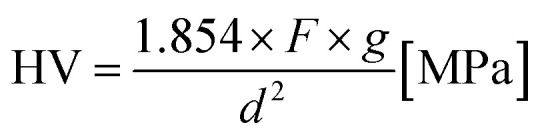
where: *F* is the force (in unit kgf, or equivalent kg) applied to the sample; *g* is gravitational constant (9.81 m s^−2^); *d* is the average length of the two diagonals of the indentation (mm).

#### Fourier transform infrared spectroscopy

Fourier transform infrared (FTIR) spectroscopy (FT/IR-4600, JASCO) was performed in the range of 500 to 4000 cm^−1^ to analyze surface functional groups and chemical bonds. The varistor surface was deeply ground using a water polishing technique, washed and dried prior to FTIR recording.

#### Electron microscopy

Surface morphology of ZnO nanoplates was observed *via* field emission scanning electron microscopy (FESEM, JEOL 7600F, Japan) instrument. The nano-ZnO powder was uniformly sprayed and adhered to a conductive carbon tape. The surface and cross-section of ZnO based varistor samples were observed *via* a scanning electron microscopy (SEM, JEOL JMS-6510LV, Japan) instrument. The average grain size (*d*) was determined by the linear intercept method, as eqn (4) and the illustration in Fig. S2†^22^4
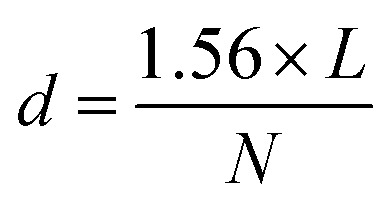
where *L* is the length (in μm unit) of a line across the micrograph; and *N* is the number of the counted grains across the drawn line (or the number of intercepts between the drawn line and the boundaries of grains; the constant of 1.56 is the experimental coefficient for the correction. The average grain size was averaged from at least 10 lines for each micrograph. For example, if the length of a line is 25 μm, the number of counted grains is 12, then *d* = 3.25 μm.

#### X-ray diffraction

Powder X-ray diffraction (XRD, Bruker Advance D8, CuK_α_ radiation, *λ* = 0.15406 nm) was used to determine the phase and crystal structure. The crystallite size was estimated by using Debye–Scherrer equation (eqn (5)) with the strongest intensity peak:5
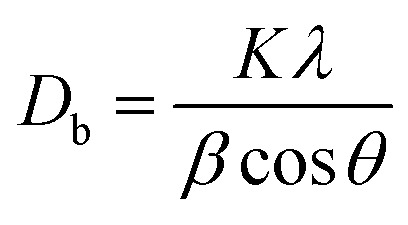
where *D*_b_ is an average crystallite size, *K* is Scherrer constant (0.94), the symbol *λ* represents the wavelength of the X-ray radiation (0.15406 nm), while *β* and *θ* are the full width at half maximum and the angle of diffraction corresponding to the strongest peak, respectively. In the XRD pattern, the (101) plane exhibited the highest intensity, which means that it is prominent in the crystalline structure.^14,23^

#### DC and AC electrical properties

The volt-ampere characteristic of a varistor was conduct on a AC/DC GPT-9802A electrical safety tester (China) with a voltage ramp of 50 V s^−1^, limit current was set at 10.99 mA for 2 seconds (the test is stopped when the limit current is reached and hold for another 2 seconds). Data (*U*, *I*) were collected at every 100 ms from the start to the end of the measurement by using a serial port utility software (version 9.3.8, Althon Studio, China). The GPT-9802A can measure the current through the sample with an accuracy of 0.01 mA (at the current limit setting of 10.99 mA). In addition, a picoammeter (Keithley 2410, USA) was still connected in series to measure the current for higher accuracy as well as evaluation of the DC leak current (at low voltage). For a standard evaluation, applied electric field was calculated as the ratio of applied voltage (*U*) over height (*h*) of the varistor sample (*E* = *U*/*h*, V mm^−1^), current density was calculated as the ratio of measured current (*I*, mA) over effective area (*S*, cm^2^) of the electrode system (*J* = *I*/*S*, mA cm^−2^). The effective diameter of the electrode was determined according to ASTM D257-14 (IEC60093) using the formula *D*_ef_ = *D* + 0.883 × *h*,^24,25^ where *D* is the actual diameter of the silver-coated electrode. The referent electric field (*E*_1mA_) or varistor threshold voltage, or breakdown threshold (*E*_b_) was taken at the value *E* corresponding to current density of 1.0 mA cm^−2^ in a *J*–*E* curve. The DC leakage current density (*J*_L_) was interpolated at *E*_L_ = 0.5 × *E*_1mA_ value from *E*–*J* curve. The non-linear coefficient (*α*) is calculated *via*eqn (6), where *J*_1_ and *J*_2_ are the current densities of 1 and 10 mA cm^−2^; and *E*_1_ and *E*_2_ are the corresponding electric fields, respectively.6
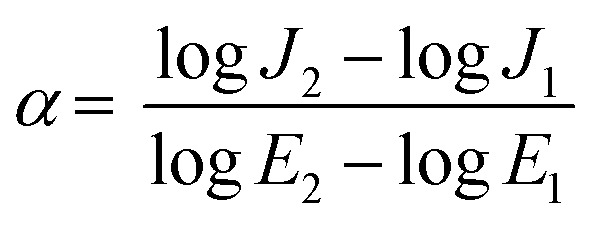


AC electrical properties (impedance and resistivity at low AC voltage) of the varistors were conducted on a VSP-300 multichannel potentiostat (EC-Lab VSP300, France), in the frequencies (50 mHz–100 kHz) with AC peak voltage of 2.0 V, and at room temperature. Varistor samples were coated with silver with the same diameter in both sides. All samples were individually mounted in the same test fixture (16451B Keysight, type D for thin film electrode, USA). Agilent E4980A Precision LCR meter was used to measure the impedance of ZnO based varistor samples at high frequencies of 1 and 2 MHz, the peak voltage applied of 2.0 V, and the same 16451B Keysight test fixture. The results were appended together and analyzed by using the EC-lab software V.11.5.

## Results and discussion

### ZnO characterization

#### SEM morphology

**Fig. 1:**
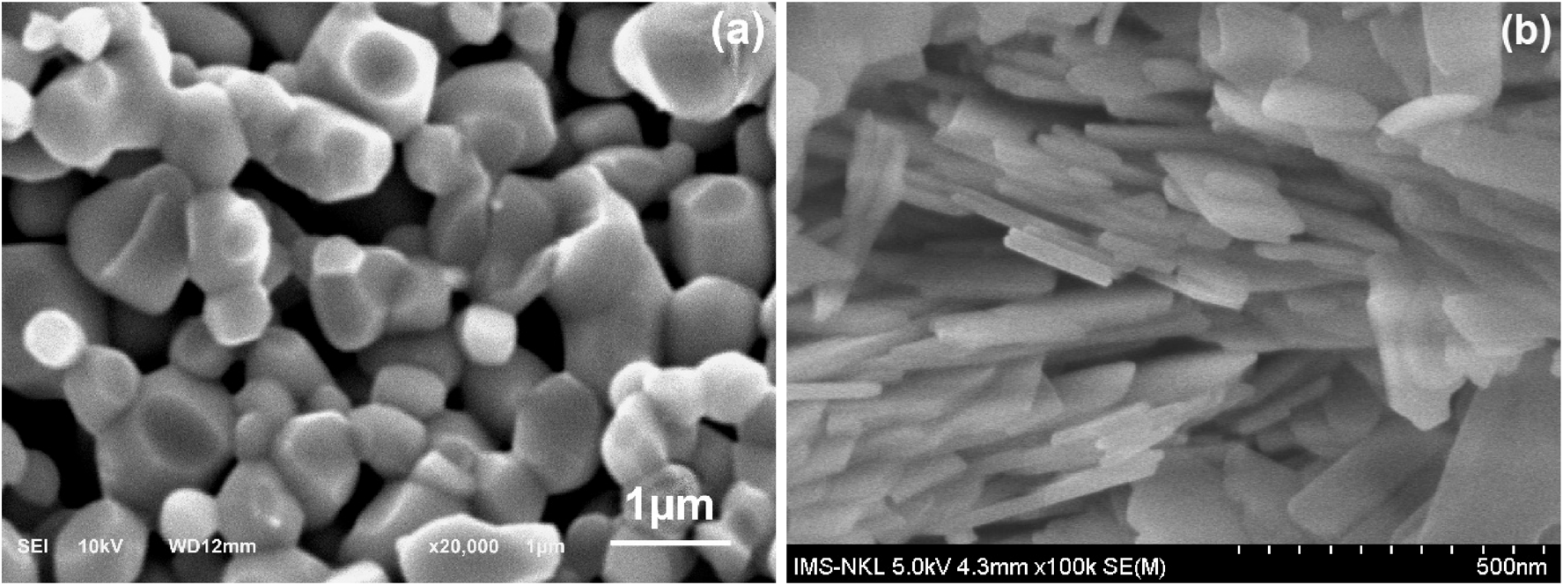
SEM images of (a) ZnO microparticles and (b) ZnO nanoplates.

Fig. 1 presents the SEM images of the micro-ZnO particles and nano-ZnO particles after calcination at 600 °C for 2 hours in a furnace. Fig. 1a shows that the particle size of micro-ZnO are in the range from 200 nm to 2 μm. Some particles are observed as clusters, each composed of 2 to 5 or more primary submicron crystals agglomerate in clusters, each consists from 2 to 5 (or more) primary particles in submicron crystals. The average particle size of micro-ZnO is about 1 μm.

Fig. 1b clearly shows the plate-like morphology of ZnO nanoparticles, characterized by their thin structure, hence referred to as ZnO nanoplates. The average thickness of ZnO nanoplates is only about 15 nm and their lengths are in the range of 100–400 nm. The formation of ZnO nanoplates was explained by Sahu *et al.*^26^ as resulting from the slow addition of OH^−^ into a Zn^2+^ solution. In this study, the hydrothermal process was conducted using urea at pH 5. This indicates that the initial concentration of [OH^−^] was very low, leading to slow reaction between Zn^2+^ ions with (OH^−^) to form Zn(OH)_2_ flakes. During hydrothermal process, OH^−^ ions were generated from the thermal decomposition of urea at high temperature and in a closed reactor (CH_4_N_2_O → CO_2_ + NH_3_ → NH_4_OH). These Zn(OH)_2_ flakes were partially converted to ZnO during hydrothermal process and fully converted to ZnO after calcination, resulting in the formation of the ultra-thin ZnO nanoplate structure. The plate-like morphology of ZnO nanoparticles is a key factor in the utilization of ZnO nanoplate powder for preparing ZnO-based varistors, with the expectation of achieving excellent electrical properties.^27^

#### FTIR and XRD analyses

**Fig. 2:**
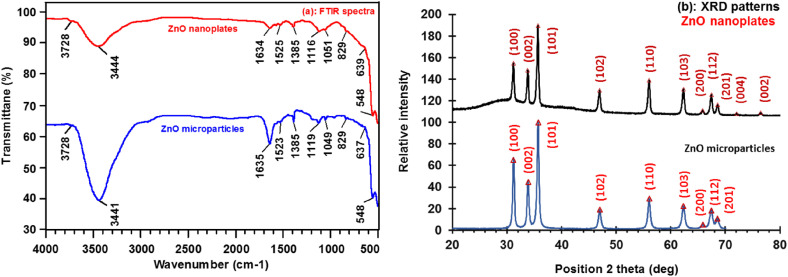
(a) FTIR spectrum and (b) XRD patterns ZnO nanoplates and microparticles calcined at 600 °C for 2 h in air.

Fig. 2 shows the FTIR spectrum and XRD diagram of ZnO nanoplates calcined at 600 °C for 2 h in air medium, respectively. It is worth to note that the FTIR and XRD spectra of micro-ZnO are quite similar to those of ZnO nanoplates. In Fig. 2a, the strong peak appears at 545 cm^−1^ is assigned for the stretching vibration of Zn–O bonds. Its high intensity means the major content of ZnO. In addition, a broad peak in the region from 3000–3750 cm^−1^ and a sharp peak at 1634 cm^−1^ are attributed to the stretching and bending vibrations of hydroxyl groups on the surface of ZnO particles. The peak at 3728 cm^−1^ is attributed to the stretching vibration of ZnOH bonds, and its weak intensity corresponds to a small amount of ZnOH bonding. The peaks 1525, 1116, 1051 cm^−1^ are attributed to specific vibrations of carbonyl groups, which indicates the presence of CO_2_ absorbed into the gallery between ZnO particles.^28–30^ The weak peak at 1385 cm^−1^ corresponds to the vibration of CO_3_^2−^ groups, due to ZnO can react with absorbed CO_2_ to form ZnCO_3_ with a trace amount.^28,30^

Fig. 2b shows the X-ray diffraction (XRD) pattern of the ZnO nanoplates. It can be seen the diffraction peaks at 2*θ* positions of 31.77°, 34.42°, 36.25°, 47.53°, 56.60°, 62.86°, 66.38°, 67.98°, 69.1°, 72.58°, and 76.95°. These peaks are fitted well with the hexagonal wurtzite ZnO (JCPDS No. 00-036-1451), corresponding to its lattice planes (100), (002), (101), (102), (110), (103), (200), (112), (201), (004), and (202), respectively. This also reveals a well-defined crystalline structure of ZnO, and confirms the high purity of the ZnO phase without any secondary phase. By using eqn (1), the crystallite size value is calculated to be about 0.9 nm. Fig. 2 also shows that the FTIR and XRD spectra of micro-ZnO are quite similar to those of ZnO nanoplates.

### Properties of as-synthesized nano-ZnO-based varistors

#### Shrinkage, density and hardness properties

Table 1 presents the axial and radial shrinkages, density, and hardness properties of the micro- and nano-ZnO-based varistors. The results in Table 1 shows that the radial and axial shrinkages of ZnO based varistors are in the range from 17.5–17.9% and 15.5–16.1%, respectively, and trend to increase as increasing sintering temperature. The shrinkage in axial height direction of a varistor is lower than that in radial direction. This is due to the fact that the green ceramic varistor is more compacted in the axial height direction than in the radial direction when pressing the powder in a cylindrical die.^31^ The density of the ZnO based varistors are in the range from 5.56–5.38 g cm^−3^, and tends to decrease when sintering temperature is exceeds 1100 °C. This phenomenon is attributed to the entrapment of insoluble gas in the pores as the liquid phase solidifies during the cooling stage and the loss of volatile Bi_2_O_3_ at high temperature.

**Table 1:** Shrinkage, density and hardness properties of the micro- and nano- ZnO-based varistors

Sample	Sintering temperature (°C)	Diametrical shrinkage (%)	Axial shrinkage (%)	Density *ρ* (g cm^−3^)	Vicker hardness (HV)
MicroZ1100	1100	17.5 ± 0.1	15.5 ± 0.2	5.49 ± 0.02	241 ± 2
NanoZ1000	1000	17.4 ± 0.2	15.6 ± 0.2	5.52 ± 0.08	255 ± 4
NanoZ1100	1100	17.5 ± 0.1	15.8 ± 0.3	5.56 ± 0.04	280 ± 5
NanoZ1200	1200	17.9 ± 0.1	16.1 ± 0.5	5.38 ± 0.04	244 ± 4

#### XRD diagrams

In comparison with MicroZ1100, the NanoZ1100 samples exhibits the higher Vickers hardness (280 ± 5 HV *vs.* 241 ± 2 HV of MicroZ1100 sample), which can be attributed to its nano size of ZnO particles. Their higher specific surface area allows the additives to distribute more effectively during sintering, enhancing the adhesion between particles within the bulk material, highlighting the effect of ZnO particle sizes. For the nano-ZnO-based varistor series, the hardness of the varistors reaches the maximum value of 280 HV at sintering temperature of 1100 °C, and tends to decrease when sintering temperature is larger than 1100 °C. This can be explained by the growth of ZnO grain size at higher sintering temperatures of 1200 °C.^32,33^ Furthermore, the formation of new phases between ZnO grains can also contribute to reducing the interaction between ZnO grains. These phenomena will be eluminated through XRD analyses and SEM morphology in the next sections.

**Fig. 3:**
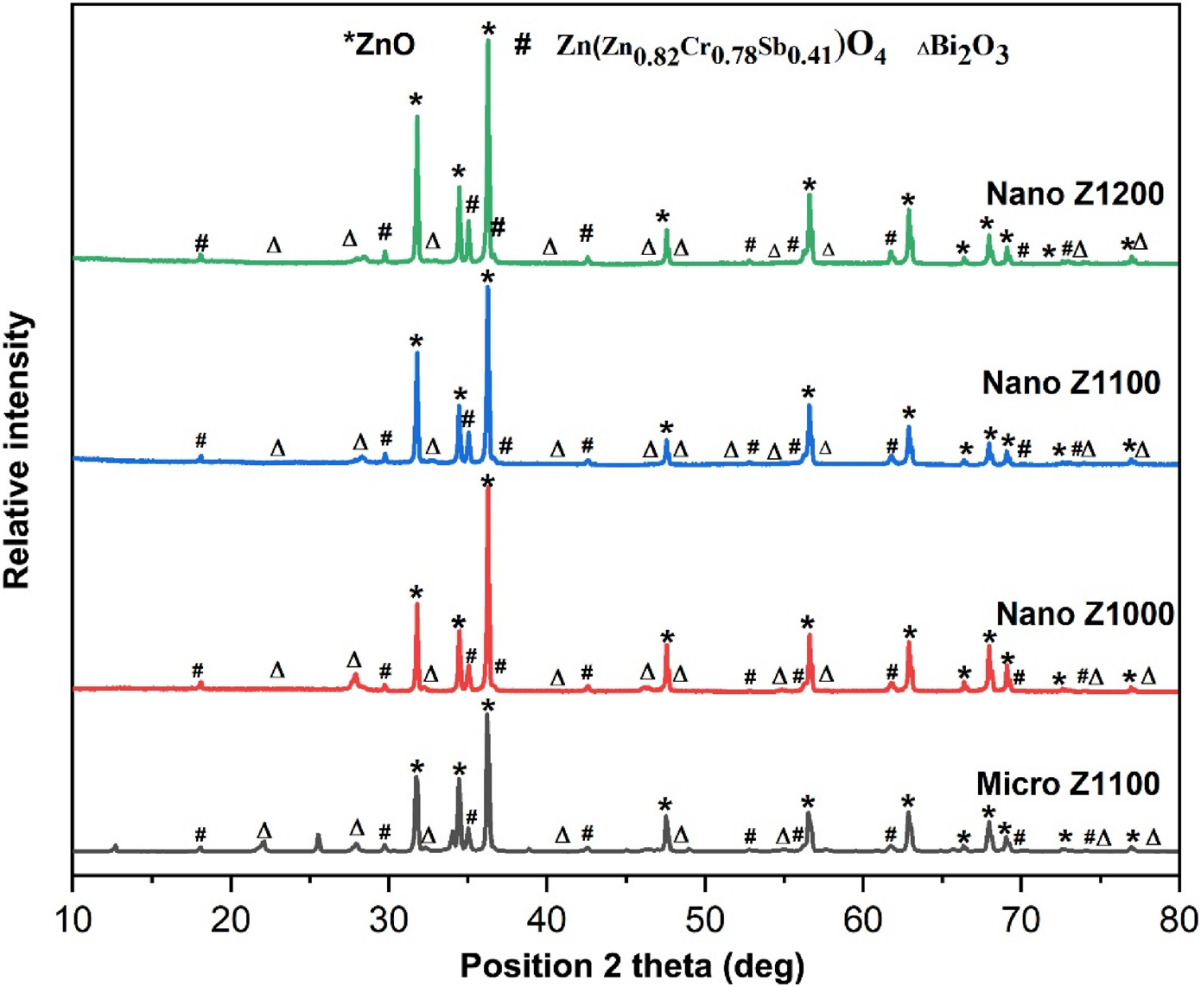
XRD patterns of ZnO based varistor ceramics sintered at 1000, 1100, and 1200 °C for 2 h.

The XRD patterns of both micro- and nano-ZnO-based varistor samples (Fig. 3) shows the typical composition of wurtzite ZnO (*) and Bi_2_O_3_ (Δ) phases, along with the modified spinel Zn_1.82_Cr_0.78_Sb_0.41_O_4_ or Zn(Zn_0.82_Cr_0.78_Sb_0.41_)O_4_ (#) phase. The ZnO phase, serving as the primary matrix, dominates the samples. Its three main diffraction peaks can be observed at 2*θ* angles of 31.78°, 34.45°, and 36.27° (±0.007°), corresponding to the lattice planes (100), (002), and (101) of the hexagonal wurtzite ZnO structure (JCPDS No. 00-036-1451). The Bi_2_O_3_ phase in cubic crystalline (JCPDS No. 01-074-1373) is also identified, with its first three peaks appearing at 22.11, 27.91 and 32.25 °C (±0.03°). Additionally, the Bi-rich tetragonal crystalline phase of Bi_2_O_3_ (γ-Bi_2_O_3_, JCPDS No. 00-018-0244) is detected in the XRD spectra of the four varistor samples, though with lower intensity.

The spinel phase, Zn_1.82_Cr_0.78_Sb_0.41_O_4_ (#, Zinc Chromium Antimony Oxide, JCPDS No. 01-082-1102), becomes more prominent at higher sintering temperatures, with its characteristic peaks appearing around the 2*θ* angles of 18.50°, 29.76°, 35.04° (±0.01°). In this study, the molar ratio Sb_2_O_3_/Bi_2_O_3_ = 1, therefore, the spinel phase mainly forms through the decomposition from the pyrochlore phase, as reaction eqn (7). The presence of Cr_2_O_3_ prevents the formation of pyrochlore as well as the formation of conventional spinel Zn_7_Sb_2_O_12_, because, it can take part in the formation of modified spinel Zn_1.82_Cr_0.78_Sb_0.41_O_4_ phase, as reaction eqn (9).^2,34,35^ This reaction may simultaneously inhibits the formation of spinel Zn_7_Sb_2_O_12_ phase (reaction eqn (8)). At 1200 °C (Nano Z1200), the peak intensity of modified spinel phase increases, suggesting a complete transition from the pyrochlore phase to the new modified spinel phase (Zn_1.82_Cr_0.78_Sb_0.41_O_4_), as reaction eqn (9).^35^

7 3Bi_2_O_3_ (liq) + 3Sb_2_O_3_ (s) + ZnO (s) + 2O_2_ → 2Zn_2_Sb_3_Bi_3_O_14_ (pyrochlore)

8 2Zn_2_Sb_3_Bi_3_O_14_ + 17ZnO → 3Zn_7_Sb_2_O_12_ (spinel) + 3Bi_2_O_3_ (liq)

9 Zn_2_Sb_3_Bi_3_O_14_ + 3.46ZnO + 1.17Cr_2_O_3_ → 3Zn_1.82_Cr_0.78_Sb_0.41_O_4_ + Bi_2_O_3_ + Sb_2_O_3_ + 1.485O_2_

#### Surface and cross-section morphology

**Fig. 4:**
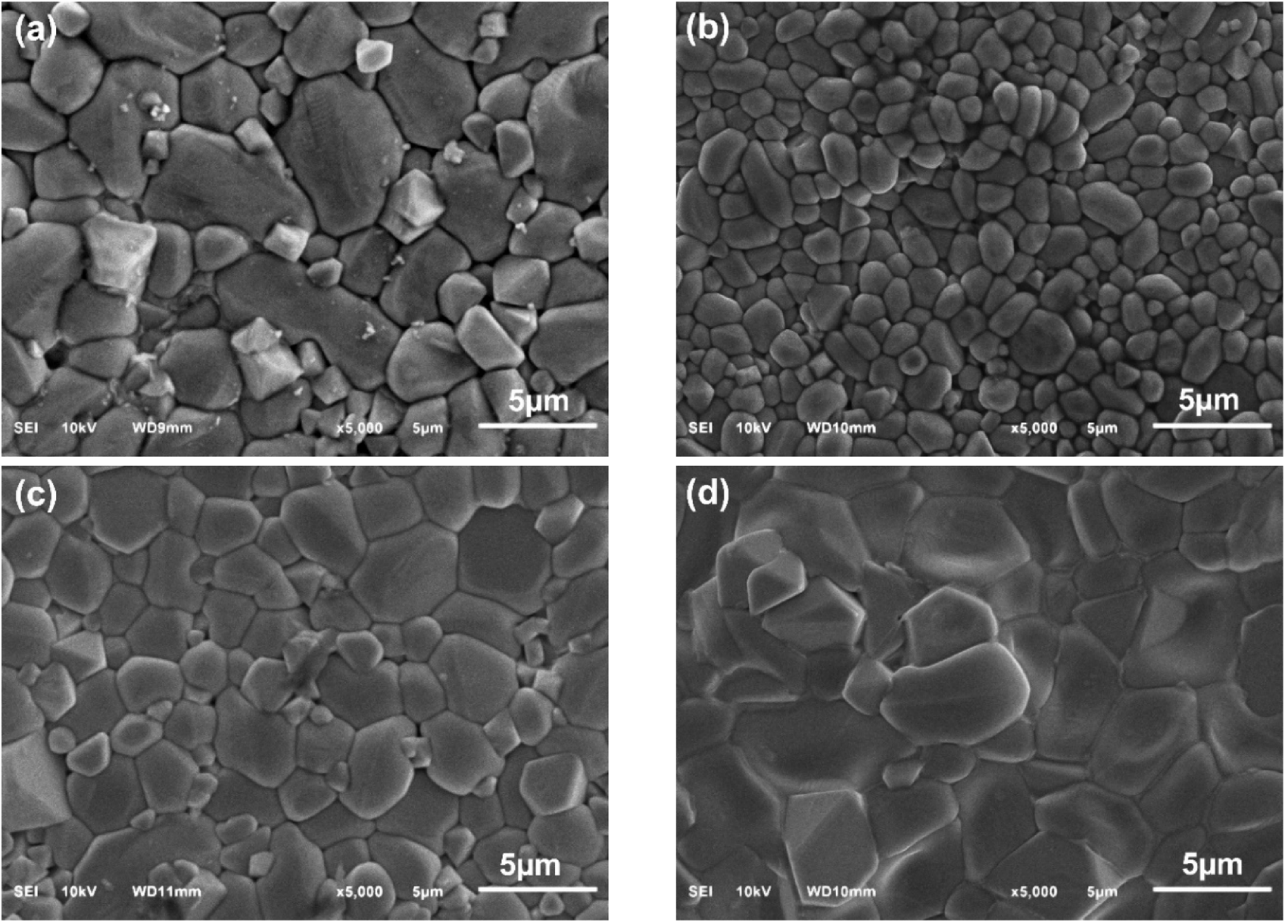
Surface SEM images of (a) MicroZ1100, (b) NanoZ1000, (c) NanoZ1100, (d) NanoZ1200 varistor.

Fig. 4 displays the surface morphological SEM images of micro- and nano-ZnO-based ceramic varistors. In this case, the SEM observation was conducted on the surface of the varistors without grinding or polishing. The average grain sizes of the varistor samples were evaluated by linear intercept method for both on-surface and cross-section, the results are shown in Table 2. The average grain size of micro-ZnO-based varistors sintered 1100 °C was 4.7 ± 0.6 μm, meanwhile, that of nano-ZnO-based varistors sintered 1100 °C was much smaller of 3.8 ± 0.3 μm. This reduction in grain size can be easy understood as smaller size of raw ZnO used to prepare varistor. The surface grain size of nano-ZnO-based ceramic varistors increases from 1.7 ± 0.2 μm at 1000 °C (NanoZ1000) to 3.5 ± 0.3 μm at 1100 °C (NanoZ1100) and 5.5 ± 0.6 μm at 1200 °C (NanoZ1200).^36^ In comparison, the MicroZ1100 sample sintered at 1100 °C (MicroZ1100) also exhibits a smaller cross-section grain size (4.8 ± 0.9 μm) than that of the NanoZ1100 at the same temperature, indicating the influence of the initial size of ZnO on grain growth. As shown in Table 2, the standard deviation of grain size in NanoZ1100 (±0.3 μm) is significantly smaller than that of MicroZ1100 (±0.9 μm), indicating a more homogeneous microstructure. This uniformity, combined with effective sintering at lower temperatures (*e.g.*, NanoZ1000), makes nano powders highly beneficial for controlling microstructure and optimizing electrical performance in varistor applications.

**Table 2:** Grain size of micro- and nano-ZnO-based varistors

Sample	Sintering temperature (°C)	Grain size on surface (μm)	Grain size in cross-section
NanoZ1000	1000	1.7 ± 0.2	1.8 ± 0.3
NanoZ1100	1100	3.5 ± 0.3	3.8 ± 0.3
NanoZ1200	1200	5.5 ± 0.6	6.8 ± 1.0
MicroZ1100	1100	4.7 ± 0.6	4.8 ± 0.9

The SEM images of the surface of the nano-ZnO-based varistors also show the changes in the shape of ZnO grains at three sintering temperature levels. At 1000 °C, the ZnO grains appear nearly round or oval. In contrast, at sintering temperatures of 1100 °C and 1200 °C, the ZnO grains exhibit polyhedral shapes, although the raw ZnO particles initially resemble nanoplate-like structures.^37^ Since the initial shape of ZnO microparticles is polyhedral, the final ZnO grains are also observed as polyhedral, but with a larger size.

**Fig. 5:**
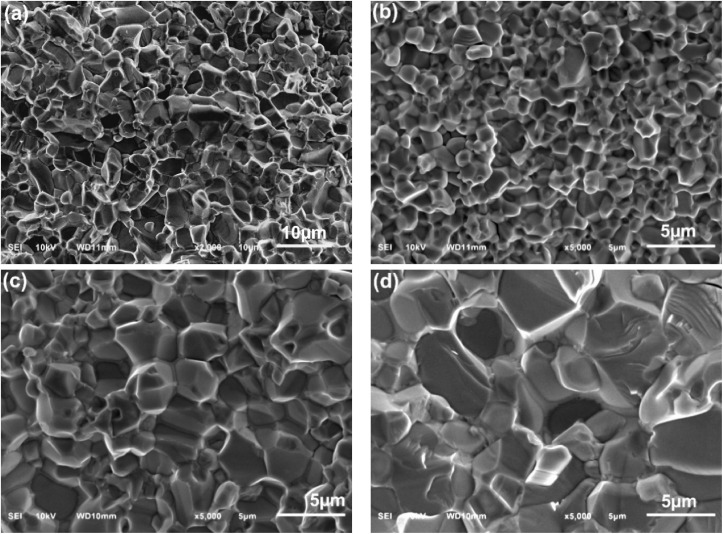
Cross-section SEM images of (a) MicroZ1100, (b) NanoZ1000, (c) NanoZ1100, (d) NanoZ1200 varistor samples.

Fig. 5 and S3 (ESI)† displays the cross-section morphology of micro- and nano-ZnO based varistor. The summarized results in Table 2 show that the ZnO grain size also increases with sintering temperature in the varistor system that uses ZnO nanoplates as the main raw material. At the same sintering temperature of 1100 °C, the average grain size of the NanoZ1100 varistor sample is 3.3 ± 0.3 μm, significantly smaller than the average grain size of the varistor sample using micro-ZnO raw material (4.8 ± 0.9 μm). The grain size in fracture-surface of varistor samples using ZnO nanoplates also increases from 2.6 ± 0.3 μm to 6.8 ± 1.0 μm as the sintering temperature rises from 1000 to 1200 °C. Unlike the surface morphology, Fig. 5 shows that the grain boundary regions between two ZnO grains in the varistor samples can be clearly observed, especially when viewed at higher magnification (the images on the left of Fig. 5). These observations confirm the hypothesis that sintering temperature and powder type (micro *vs.* nano) are critical factors in microstructural evolution. In NanoZ samples, grain growth is significantly suppressed at lower temperatures due to the presence of modified spinel particles, which act as pinning agents to stabilize grain boundaries and prevent coarsening. However, at higher temperatures (1200 °C), the enhanced grain growth indicates a diminished pinning effect and increased atomic mobility, promoting grain enlargement.^38^

#### FTIR spectra

**Fig. 6:**
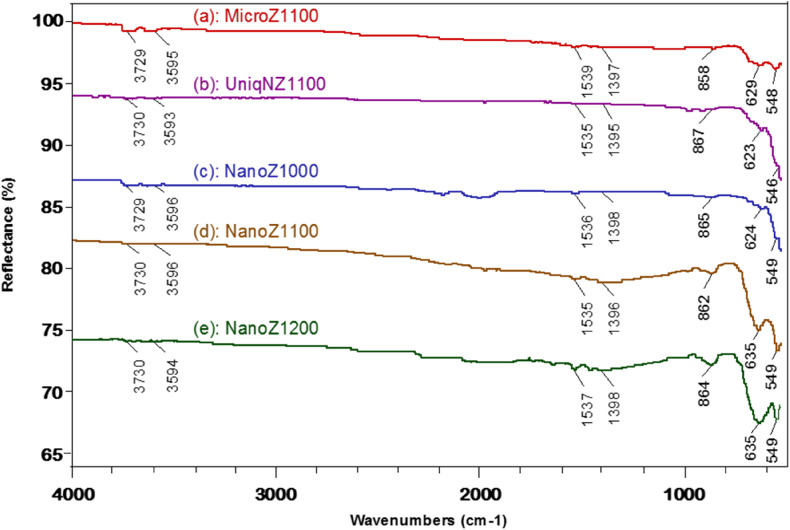
FTIR spectra of (a) micro-ZnO-based varistor, (b) unique nano-ZnO varistor sintered at 1100 °C, and nano-ZnO-based varistors sintered at (c) 1000, (d) 1100 and (e) 1200 °C.

Fig. 6 displays the FTIR spectra of unique nano-ZnO varistor, micro-ZnO-based varistor sintered at 1100 °C, and nano-ZnO-based varistors sintered at 1000, 1100 and 1200 °C. It is noted that the micro-ZnO and nano-ZnO-based varistors were made with the same compositions, and unique nano-ZnO varistor was only made from 100% of ZnO nanoplates. Fig. 6 shows that the intensities of stretching and bending absorptions of OH groups are relatively low, indicating the progressive release of the most surface moisture and OH groups of ZnO particles during sintering process at high temperature. The bands at 1535–1539 cm^−1^ and 1380–1397 cm^−1^ are attributed solely to the re-adsorption of CO_2_ on the surface of the ZnO-based varistors, because the pre-adsorbed CO_2_ and CO_3_^2−^ have been theoretically released or degraded after sintering process. Due to the predominant ZnO component in the varistors, the peaks observed in the ranges of 858–867, 546–549 cm^−1^ can be attributed to stretching vibrations of ZnO bonds, the peaks in the region of 623–635 cm^−1^ correspond to stretching vibration of ZnO within the crystalline structure.^39^ It is also seen shows that the nano-ZnO-based varistors samples (NanoZ1100, and NanoZ1200) exhibit sharper and more prominent ZnO peaks, highlighting enhanced crystallinity. This finding demonstrates that FTIR analysis should be employed in the characterization of ZnO-based varistors.

### Volt-ampere characteristic curves and DC electrical properties

**Fig. 7:**
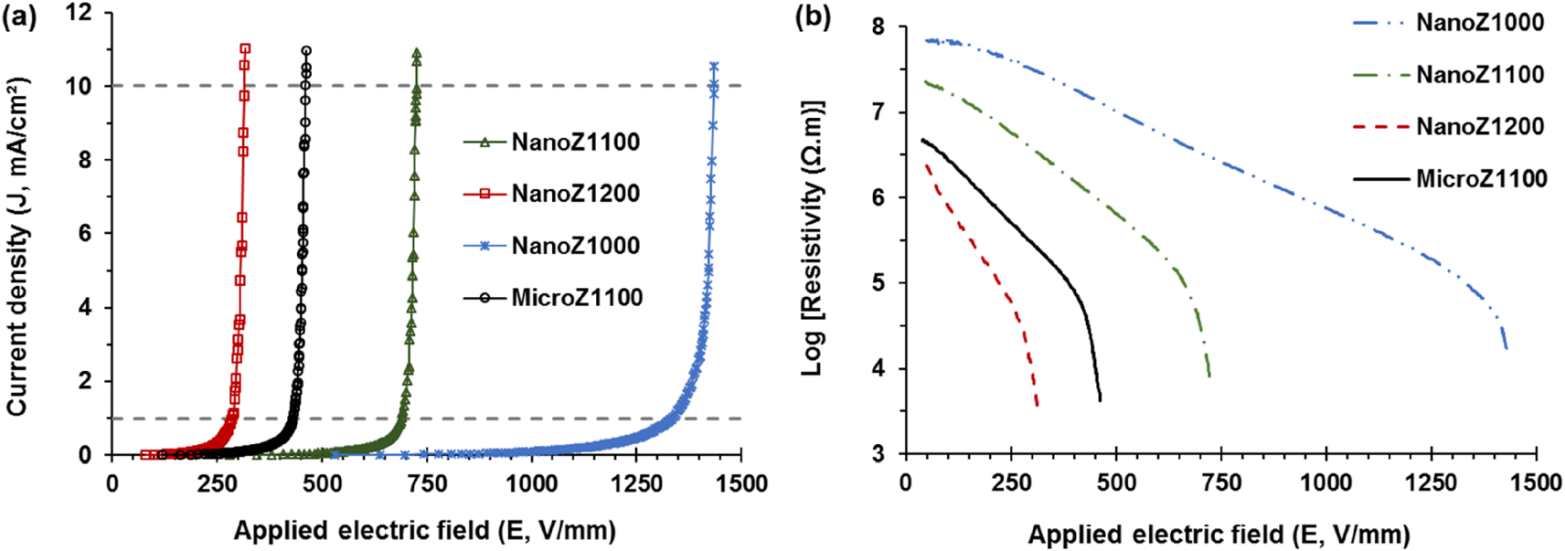
The voltage-ampere characteristic curves of ZnO based varistors sintered at different temperatures, (a) *E*–*J* curves; (b) *E*–log(resistivity) curves.

Fig. 7 presents the DC volt-ampere characteristic curves, illustrating the density current (*J*, mA cm^−2^) as a function of applied electric field (*E*, V mm^−1^), along with the logarithm of DC resistivity (*ρ*, MΩ m) as a function of applied *E*. The data include micro-ZnO-based varistor sintered at 1100 °C, and nano-ZnO-based varistors sintered at 1000, 1100 and 1200 °C. The breakdown threshold (*E*_b_, V mm^−1^), breakdown threshold per grain boundary (*V*_gb_, V), nonlinear coefficient (*α*), and leakage current density (*J*_L_, mA cm^−2^) parameters determined from the *E*–*J* curves are summarized in Table 3.

**Table 3:** Electrical parameters of varistors prepared with different at different temperatures

Sample	*υ*_gb_ (V)	*E*_1mA_ (V mm^−1^)	*α*	*J*_L_ (μA cm^−2^)
NanoZ1000	2.41	1336	34.3	11.9
NanoZ1100	2.62	689	48.5	9.7
NanoZ1200	1.94	286	24.6	27.6
MicroZ1100	2.07	432	35.8	23.4

Fig. 7a shows that all above varistors have two distinct characteristic regions. In the first region (*E* < *E*_1mA_), the behavior is approximately linear behavior (*α* < 10) at low electric fields. In the second region (*E* > *E*_1mA_), the varistors demonstrate the nonlinear behavior. The nonlinear behavior of the nano-ZnO-based varistors depends on both sintering temperature and the particle size of raw ZnO material. As the sintering temperature increases, the breakdown threshold (*E*_b_ = *E*_1mA_) tends to decrease, suggesting grain growth and a reduction in the number of grain boundaries per unit length.

The nonlinearity coefficient (*α*) exhibits a significant variation, with the highest value observed for the sample sintered at 1100 °C (*α* = 48.5), indicating optimal grain boundary characteristics for nonlinear behavior. The breakdown threshold and breakdown threshold per grain boundary of NanoZ1100 (689 V mm^−1^, 2.62 V) are significantly higher than those of MicroZ1100 (432 V mm^−1^, 2.07 V). This indicates that the nano-ZnO-based varistor has a finer grain structure, resulting in more grain boundaries per unit length, which enhances the breakdown voltage.

Additionally, the leakage current density of NanoZ1100 (9.7 μA cm^−2^) is lower than that of MicroZ1100 (23.4 μA cm^−2^). This lower leakage current indicates that the grain boundaries in NanoZ1100 varistors provide more effective barrier potential, reducing undesired current flow under low-field conditions.

Fig. 7b also shows that the resistivity of the varistor samples is initially very high, around 10^6.5^–10^8^ Ω m, corresponding to the insulating state. As the applied electric field (*E*) increases, the resistivity decreases rapidly. However, a sharp drop in resistivity occurs after reaching the breakdown electric field *E*_1mA_, where the varistor transitions from a high-resistance state to a highly conductive state. In the region with *E* > *E*_1mA_, the resistivity decreases dramatically to around 10^3^–10^4^ Ω m. This sharp decline marks the transition from the pre-breakdown region to the highly conductive state, where the varistor begins to exhibit strong nonlinearity and efficient voltage clamping behavior. The resistivity behavior of the nano-ZnO varistors sintered at different temperatures (1000, 1100, and 1200 °C) shows a clear difference. The NanoZ1000 sample exhibits the highest resistivity across all electric field ranges, indicating a more insulating nature. As the sintering temperature increases to 1100 °C (NanoZ1100) and 1200 °C (NanoZ1200), the resistivity decreases, suggesting enhanced grain growth and improved electrical conductivity.

Comparing the micro-ZnO varistor (MicroZ1100) and nano-ZnO varistor (NanoZ1100), both sintered at 1100 °C, the MicroZ1100 sample shows lower resistivity than its nano counterpart at lower electric fields. However, as the electric field increases, the resistivity of NanoZ1100 decreases more rapidly, indicating a stronger nonlinearity and better varistor performance. This suggests that nano-structured ZnO enhances the breakdown characteristics and nonlinear behavior compared to micro-structured ZnO.

The results presented in Table 3 can be explained as follows. At a sintering temperature of 1000 °C, limited dopant diffusion, small ZnO grain sizes, and narrow grain boundary layers are observed. This leads in a high number of grain boundaries per unit length and a high number of depletion regions at the grain boundaries, resulting in an increase in grain boundary resistivity.^40,41^ However, the varistor effect, indicated by a nonlinear coefficient (*α*) of 34.3, remains relatively low, although a high breakdown voltage and resistivity can be achieved.

As the sintering temperature exceeds 1100 °C, ZnO grains continue to grow, forming thicker and more distinct grain boundary layers on the micrometer scale. This grain growth reduces the number of grain boundaries per unit length and decreases the resistivity of the grain boundary layer,^18,19^ ultimately lowering the varistor effect (*α* = 24.6 at a sintering temperature of 1200 °C). Similarly, when using micro-sized ZnO as the raw material, its lower specific surface area causes dopants to be distributed over a smaller area, promoting the formation of thicker grain boundary layers.

As sintering progresses at 1100 °C, ZnO grains continue to grow from the micrometer scale, further increasing grain size. Consequently, in the MicroZ1100 sample, the larger ZnO grains lead to more pronounced and thicker grain boundary layers. This results in lower overall resistance of grain boundaries compared to the NanoZ1100 sample, where the finer ZnO grains contribute to a higher density of grain boundaries and greater resistivity.

A sintering temperature of 1100 °C is considered optimal, as it balances grain boundary layer thickness and ZnO grain size while enabling efficient dopant diffusion into the grain boundary region. This ensures an improved electrical response without excessive grain growth, maintaining stable varistor performance. At this temperature, the highest nonlinear coefficient (*α* = 48.5) is obtained, while a moderate breakdown voltage and resistivity are still achieved.

It can be suggested that nano-ZnO-based varistors (NanoZ1100) exhibit superior electrical properties compared to micro-ZnO-based ones (MicroZ1100), including a higher breakdown field, stronger nonlinearity, and lower leakage current. Nano-ZnO particles possess a significantly higher specific surface area, which facilitates deeper dispersion of additive oxides. This results in a greater number of active sites for potential barrier formation, thereby enhancing the non-linear electrical behavior. As shown in the FESEM images, the use of nano-ZnO powders leads to varistors with fine-grained microstructures. The grains grow from the nanoscale to the microscale during sintering, resulting in smaller average grain sizes compared to those obtained using micro-ZnO powders. This finer microstructure contributes to higher breakdown voltage and improved nonlinear coefficient. These mechanisms have been observed and discussed in previous studies,^36,41^ and are consistent with the improved performance of our nano-ZnO-based varistors compared to those fabricated from micro-ZnO.

These advantages highlight the potential of nano-ZnO powders in enhancing varistor performance, making them more suitable for high-voltage and high-performance applications. Moreover, sintering temperature not only influences the structural properties but also impacts the nonlinear characteristics of ZnO-based varistors. Optimizing the sintering conditions at 1100 °C is essential to ensure the effective operation of varistors in power applications.

### Impedance and AC electrical properties

**Fig. 8:**
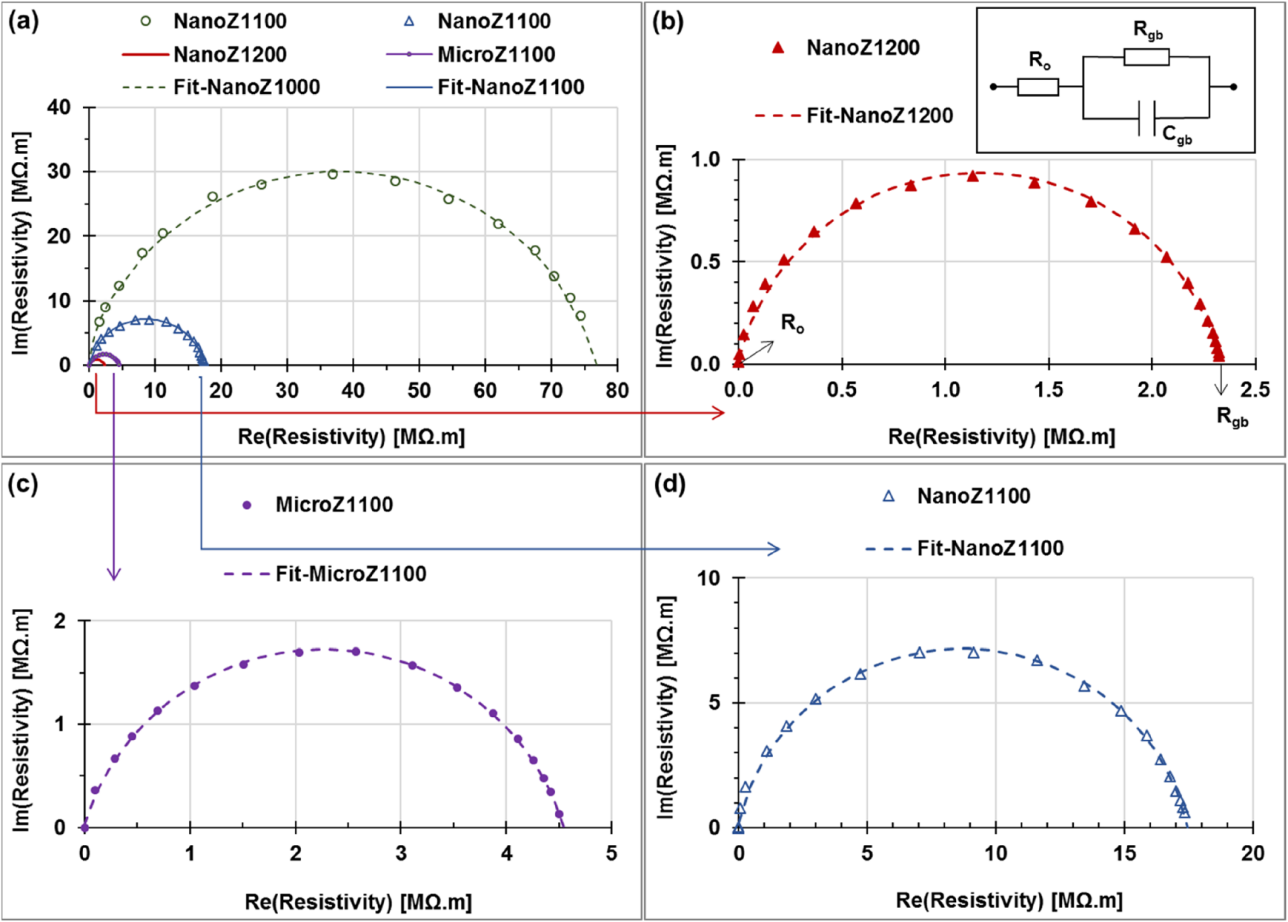
Resistivity Cole–Cole plots of ZnO based varistors sintered at different temperatures, (a) NanoZ1000 (hollow circle); (b) NanoZ1200, (c) MicroZ1100, and (d) NanoZ1100.

The Cole–Cole plots of resistivity (in units of MΩ m) for ZnO-based varistors sintered at different temperatures are displayed in Fig. 8. These plots exhibit a characteristic semicircular arc, which are fitted well with the [*R*_o_ in series (*R*_gb_//*C*_gb_)] equivalent circuit model, as illustrated at the top of Fig. 8b.^16,42^ In this model, *R*_o_ represents the ohmic resistivity of ZnO, while *R*_gb_ and *C*_gb_ correspond to the grain boundary resistivity and specific capacitance, respectively.^43^ The AC electrical impedance properties of micro- and nano-ZnO-based varistors are summarized in Table 4. The results in Table 4 indicate that the ohmic resistivity and grain boundary resistance depend on the sintering temperature and microstructure significantly.

**Table 4:** Electrical impedance properties of micro- and nano-ZnO-based varistors

Varistor sample	*R*_o_ (Ω)	*ρ*_o_ (Ω m)	*ρ* at 2 MHz (Ω m)	*ρ*_gb_ (MΩ m)	*R*_gb_ (MΩ)
NanoZ1000	1.97	0.083	11.51	76.90	1829
NanoZ 1100	1.29	0.072	5.03	17.45	312.7
NanoZ 1200	0.81	0.038	1.42	2.34	50.31
MicroZ1100	1.08	0.049	2.64	4.55	100.1

The ohmic resistance and resistivity (*R*_o_ and *ρ*_o_) show a decreasing trend as the sintering temperature increases. The highest values of *R*_o_ and *ρ*_o_ for the NanoZ1000 sample (1.97 Ω m and 0.083 Ω, respectively). Conversely, the lowest values of *R*_o_ and *ρ*_o_ for NanoZ1200 (0.038 Ω m and 0.82 Ω) suggests better electrical conductivity in the microstructure-based varistor. The moderate values of *R*_o_ (1.08–1.29 Ω) and *ρ*_o_ (0.049–0.072 Ω m) are observed for the Micro1100 and NanoZ1100 samples, respectively.

Grain boundary resistivity and grain boundary resistance (*ρ*_gb_ and *R*_gb_) also show significant variations. The NanoZ1000 sample exhibits strong grain boundary insulation, as indicated by the largest semicircle in Fig. 8a and the highest resistivity (76.90 MΩ m), corresponding to a resistance of 1829 MΩ. As the sintering temperature increases, these values decrease significantly in the NanoZ1200 sample (*ρ* = 2.34 MΩ m and *R* = 50.31 MΩ, respectively). The MicroZ1100 and NanoZ1100 samples have a moderate grain boundary resistivity values (4.55 and 2.34 MΩ m) and resistances (100.1 and 312.7 MΩ), respectively.

In comparison within nano-ZnO-based varistor series, increasing the sintering temperature significantly reduces the grain boundary resistance (*R*_gb_) and enhances electrical conductivity. The NanoZ1000 sample (sintered at 1000 °C) exhibits the largest semicircle, indicating the highest *R*_gb_ (1829 MΩ), which suggests strong impedance at grain boundaries. This is attributed to incomplete grain coalescence and insufficient diffusion at lower sintering temperatures. When the temperature is increased to 1100 °C (NanoZ1100), *R*_gb_ drops to 312.7 MΩ, indicating improved intergranular contact and enhanced charge transport. However, the most significant reduction is observed at 1200 °C (NanoZ1200), where *R*_gb_ decreases drastically to 50.31 MΩ, demonstrating the highest degree of grain growth and boundary connectivity among the nano-ZnO samples.

In the comparison between nano- and micro-ZnO at 1100 °C, it can be revealed the influence of grain size on electrical properties. The NanoZ1100 sample exhibits the lower *R*_o_ (0.81 Ω) and *R*_gb_ (50.31 MΩ) than MicroZ1100, indicating that grain enlargement effectively reduces intrinsic resistance. However, its grain boundary resistivity (*R*_gb_ = 4.55 MΩ·m) remains higher than that of NanoZ1200 (2.34 MΩ m), suggesting that nanoscale ZnO grains sintered at high temperatures yield superior electrical performance due to enhanced charge carrier mobility at grain boundaries. Higher sintering temperatures reduce grain boundary resistance, facilitating improved electrical conductivity. Therefore, the NanoZ1200 sample exhibits superior electrical conductivity and lower grain boundary resistance, making it suitable for low-voltage applications. In contrast, the NanoZ1100 sample, with its higher grain boundary resistance, can be applied in high-voltage applications where a higher breakdown field is required.

These findings indicate that NanoZ1000 exhibits the highest grain boundary resistance, making it suitable for applications requiring strong electrical insulation. In contrast, NanoZ1200 demonstrates the best electrical conductivity but reduced grain boundary insulation, which may be advantageous for applications requiring higher current conduction. MicroZ1100 and NanoZ1100 presents a balance between conductivity and insulation, however the MicroZ1100 sample does not outperform nano-based varistors in either aspect. These results highlight the influence of microstructure and sintering temperature on the electrical performance of ZnO-based varistors, providing insights into optimizing their applications in electronic devices.

**Fig. 9:**
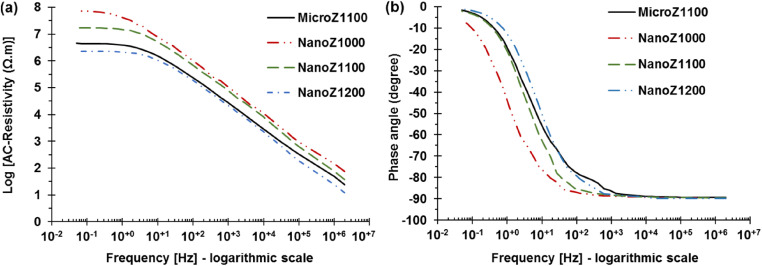
(a) The AC resistivity (Ω m) and (b) the phase angle (degree) of resistivity of as a function of frequency for ZnO-based varistors.

Fig. 9 illustrates the variation of AC resistivity and the phase angle between imaginary and real part of resistivity of ZnO-based varistors as a function of frequency. The logarithmic AC resistivity curves (Fig. 9a) exhibit a decreasing trend with increasing frequency, indicating the frequency-dependent electrical behavior of the varistors. At low frequencies (*f* < 10 Hz), the AC resistivity values are very high (≅*R*_gb_, 10^6.5^–10^8^ Ω m), and approximately equal to the DC resistivity at low applied field, due to the measurement in the nonlinear region of ZnO varistors. Among the varistor samples, NanoZ1000 exhibits the highest AC resistivity, while NanoZ1200 shows the lowest value, suggesting that the microstructure and sintering temperature significantly influence grain boundary resistance.

As the frequency increases (10^1^–10^6^ Hz), the AC resistivity of all samples converges, indicating that the contribution of grain boundary resistance diminishes, and the bulk conduction mechanism dominates. Notably, NanoZ1200 demonstrates lower resistivity compared to NanoZ1000, which aligns with the expected reduction in grain boundary resistance due to the higher sintering temperature. The phase angle plots (Fig. 9b) provide further insight into the electrical response of the varistors. At low frequencies (<1 Hz), the phase angle is close to 0°, indicating predominantly resistive behavior. As the frequency increases, the phase angle rapidly shifts towards −90°, signifying a transition from resistive to capacitive characteristics. This shift occurs more abruptly for NanoZ1000 and NanoZ1100, suggesting stronger grain boundary effects and polarization phenomena compared to NanoZ1200 and MicroZ1100.

## Conclusions

This work explores the effects of sintering temperatures on the structural, mechanical, and electrical properties of nano-ZnO varistors. By optimizing the sintering process and utilizing the appropriate dopant oxide system, significant improvements in microstructural uniformity and electrical performance have been achieved successfully for nano-ZnO varistors. Once sintered at temperatures of 1000–1200 °C, the values of their shrinkage, average grain size and hardness are 17–19%, 1.7–6.8 μm, and 200–280 HV, respectively. At the optimal sintering temperature (1100 °C), the nano-ZnO varistors achieved a balance between high nonlinearity (*α* = 48.5), low leakage current (*J*_L_ = 9.7 μA cm^−2^), and relatively high breakdown field (*E* = 689 V mm^−1^). For all samples, the values of grain boundary electrical resistivity at low frequency were similar to those of DC resistivity at the low applied electric fields (∼106.5–108 Ω m). However, at the higher frequencies, the values of AC resistivity and phase angle showed a characteristic transition from resistive to capacitive behaviors, with a more pronounced shift for NanoZ1000 and NanoZ1100 samples. These findings might provide a fundamental understanding of the structure–property relationship in ZnO varistors, which can be further optimized for specific industrial applications.

## Author contributions

Tham Do Quang: conceptualization (lead), formal analysis (equal); funding acquisition (lead); investigation (equal); methodology (equal); visualization (equal); writing – original draft (equal), review and editing (lead). Huy Nguyen Trung: methodology (equal); visualization (equal); investigation (equal), writing – original draft (lead). Trang Nguyen Van, Kieu Anh Vo Thi, Hong Cao Thi, Xuyen Nguyen Thi, Tuan Anh Nguyen (Bsc), Chinh Tran Van, Duy Lai Van: formal analysis (equal), investigation (equal); methodology (equal); visualization (equal), writing – original draft (equal). Tuan Anh Nguyen (Dr), Lam Tran Dai: review (equal). Duong Duc La: original draft, review and editing (equal).

## Conflicts of interest

The authors declare no competing interests.

## Supplementary Material

RA-015-D5RA01534K-s001

## Data Availability

The data available within the article and its ESI.†
