# Supplementary material for: Tailoring morphological and electrical properties of nanoplate-ZnO varistors via sintering temperature
Source: RSC Adv. 2025 Jun 12;15(25):20006–19. doi: 10.1039/d5ra01534k (PMC12159798; doi:10.1039/d5ra01534k)
Supplement: RA-015-D5RA01534K-s001 [file RA-015-D5RA01534K-s001.pdf]

This journal is © The Royal Society of Chemistry 2025

Supplementary material

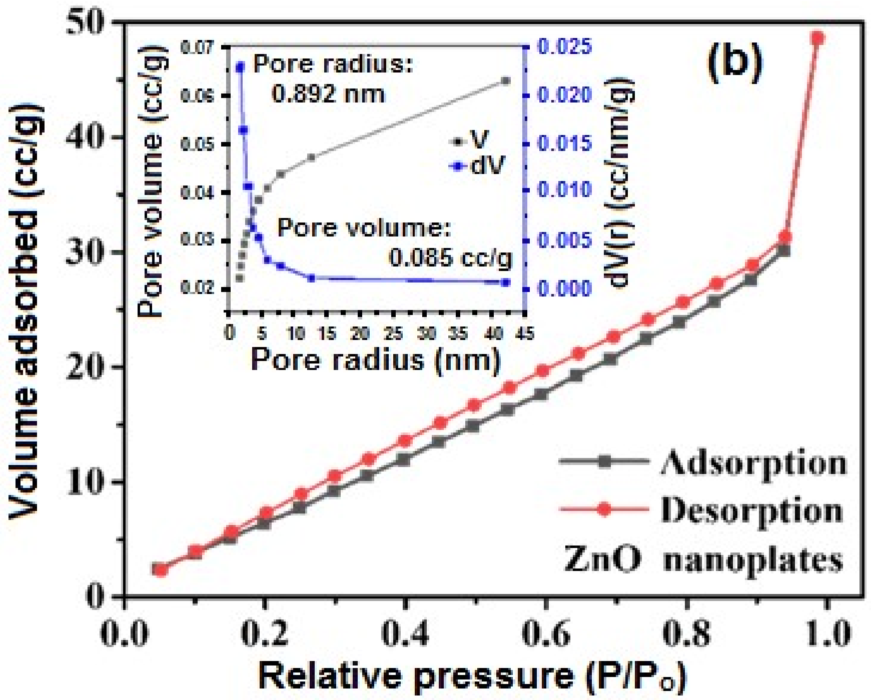

Figure S1. Nitrogen adsorption/desorption of ZnO nanoplates.

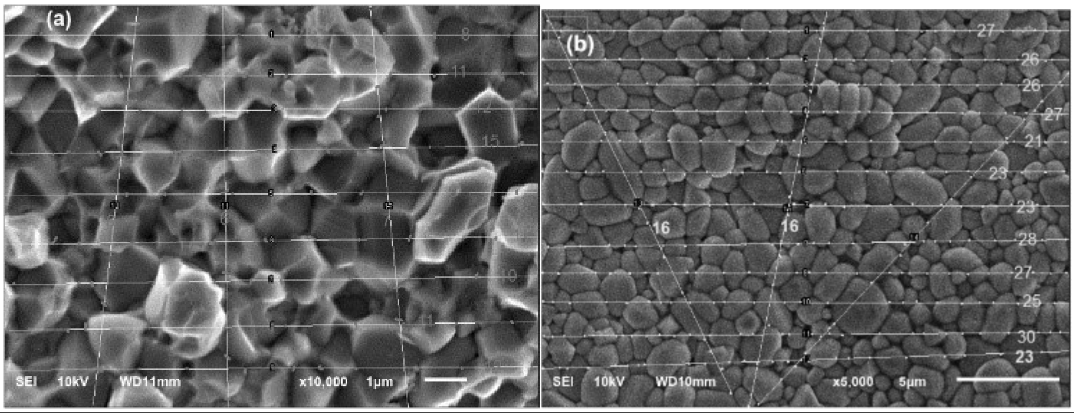

Figure S2. The illustration of linear intercept method for averaging ZnO grain size (a): on the cross-section, and (b): on the surface of varistor samples.

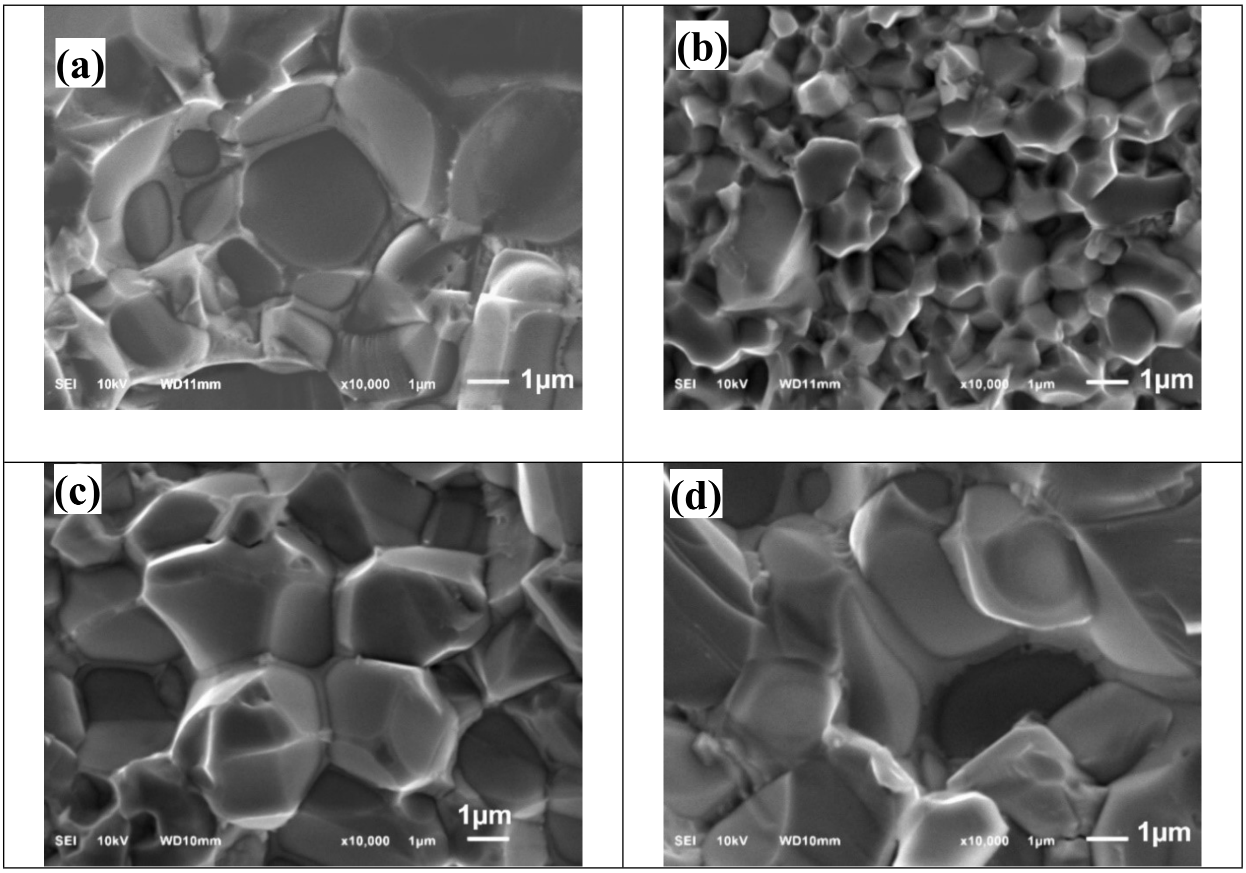

Figure S3. Cross-section SEM images of (a) MicroZ1100, (b): NanoZ1000, (c): NanoZ1100, (d): NanoZ1200 varistor samples.
